# Cytomegalovirus Infection and Its Relationship with Leukocyte Telomere Length: A Cross-Sectional Study

**DOI:** 10.1155/2021/6675353

**Published:** 2021-02-15

**Authors:** Zhu Lin, Hongmei Gao, Bing Wang, Yongqiang Wang

**Affiliations:** Department of Intensive Care Unit, Tianjin First Central Hospital, Tianjin, China

## Abstract

**Background:**

Telomeres undergo shortening with each cell division, which could be accelerated by infection. The association between virus infection and telomere length is poorly understood. In the present study, we investigated the putative associations between leukocyte telomere length (TL), cytomegalovirus (CMV) infection, and C-reactive protein (CRP) in a national representative sample of noninstitutionalized population.

**Methods:**

We analyzed data that was collected in a cross-sectional setting, where 3,987 participants were enrolled with available data on telomere length. The association between telomere length with previous CMV infection and CRP was analyzed using multivariable linear regression models. We further tested if obesity, measured by body mass index (BMI), and smoking could modify this relationship.

**Results:**

In total, around 46% percent of the study population were men and 54% were women. Average ages were 35.1 years for men and 35.0 years for women. One unit increase of CMV antibody IgG titer was associated with -0.07 (95% confidence interval: -0.12, -0.01) unit decrease of leukocyte TL when sex was adjusted for. After additionally adjusting for BMI and smoking status, the magnitude of the association was only slightly decreased to -0.06 (95% confidence interval: -0.11, -0.01). The effect sizes were comparable after additionally adjusting for CRP. These analyses imply that previous CMV infection affects leukocyte TL through pathways other than CRP.

**Conclusions:**

Previous CMV infection was associated with shorter leukocyte TL. This association was independent of CRP.

## 1. Background

Telomeres are repeated sequences of nucleotides with protecting proteins at the end of chromosomes. The length of telomeres (TL) shortens as cell divides. TL has therefore been acknowledged as a putative predictive biomarker for biological aging [1]. TL shortens as people become older and has been reported to be a risk factor for metabolic disorders like diabetes [2, 3], cardiocerebrovascular disease, and metabolic syndrome [4–7], among others like dementia and cancer [8–14]. Its association with immunological functions and virus infection has, however, rarely been investigated [15].

Chronic cytomegalovirus (CMV) infection significantly influences the immune system and has been found to be one of the main determinants of immune senescence in the elderly [16, 17]. The serum titer of CMV antibody is widely used and measured in both research and clinics as one of the biomarkers for CMV infection. However, the role of CMV infection/antibody titer in cellular senescence (e.g., biological aging as measured by leukocyte TL) has rarely been extensively examined. A landmark report on this topic was a study of 159 healthy individuals from the Netherlands, which found that telomere length shortening was even more pronounced in CMV-seropositive individuals [18]. A more recent study using longitudinal data also found that CMV infection and inflammatory biomarkers were associated with mean levels of TL [19]. Another study, however, did not find significant differences of TL in participants with CMV seropositive and negative from a cross-sectional survey [20].

Additionally, studying the putative association between TL and CMV could supply further knowledge regarding the functions of infection and immune system in biological aging. Because C-reactive protein (CRP) was in a close relationship with both TL and CMV infection, this relationship between TL and CMV infection could therefore be confounded or mediated by CRP. We hypothesized, throughout the manuscript, that higher levels of CMV antibody could be a risk factor for shorter leukocyte TL. This relationship may be also influenced by CRP. The analysis will be based on data from the National Health and Nutrition Examination Survey (NHANES).

## 2. Methods

### 2.1. Study Materials

The NHANES has been a continuous population-based survey led and conducted by the Centers for Disease Control and Prevention (CDC) with the primary aim to estimate the prevalence of various chronic disease, health status, and nutritional conditions among noninstitutionalized population across the US on a regular basis since long. The present study extracted data from the 1999-2000 and 2001-2002 cycles. During these periods, the study participants donated blood samples and other biomaterials. The leukocyte TL was also measured thereafter [21].

### 2.2. Telomere Length Measurement

The measurement procedures and standard operation process for telomere length assessment were reported elsewhere previously [21, 22]. The actual DNA processing procedure was conducted by the laboratory of the NHANES Division in the Centers for Disease Control and Prevention, USA. DNA samples were extracted from peripheral blood samples and then stored at -80°C. The TL measurement was performed later in another laboratory in UCSF. A standard quantitative polymerase chain reaction (PCR) method was employed for the assessment. This method was able to calculate the relative TL as commonly presented as the *T*/*S* ratio in previous publications. On average, 98.7% of the samples passed the quality control process.

### 2.3. Cytomegalovirus Antibody Ascertainment

The detailed procedures for the CMV antibody assessment were described on the official website (https://wwwn.cdc.gov/Nchs/Nhanes/2001-2002/SSCMV_B.htm). In short, CMV-specific IgG was measured with an ELISA method. Optical density shows whether the antibody titer was low or high.

### 2.4. Statistical Analysis

We log-transformed leukocyte TL and CRP and employed multivariable linear regression models to estimate and test the association between leukocyte TL and CMV antibody. We presented continuous variables by mean and standard deviations and category variables as the number and proportions. Different regression models were used to calculate these associations. Firstly, we estimated the crude association in which the leukocyte TL is the dependent variable and independent variables are CMV antibody, ethnicity, age, and sex. We further adjusted for body mass index (BMI) in the second model. The third model was moreover adjusted for smoking status. The fourth model was controlled for CRP. We also recorded CMV antibody and CRP to be a categorical variable by quartiles and presented the analyses as well. Because NHANES used complex survey designs, weights for the sampling were considered in the multivariable analyses using the survey package in R. We used R 3.6 for all statistical analysis and *P* < 0.05 as a statistical significance level.

## 3. Results

Table 1 presents the demographic characteristics for men and women. 3,987 participants had both leukocyte TL and CMV antibody data. 1,816 (46.0%) were men and 2,171 (54.0%) were women. Men and women had similar average ages (35.1 and 35.0 years for men and women, respectively). While women had slightly longer TL compared with men (1.13 and 1.12 T/S ratio), it is not statistically significant. CMV antibody was higher in women (1.48 and 1.09 AU/mL).

Table 2 describes the associations between CMV antibody and leukocyte TL in this study population. We first categorized CMV antibody to a categorical variable with four equal groups by quartiles and then used the categorical CMV antibody as an independent variable to investigate its association with leukocyte TL. Compared with people in the lowest CMV antibody group, people in the highest CMV antibody group had -0.18 (95% confidence interval: -0.32, -0.05) lower leukocyte TL in the first model, where age and sex were adjusted for. The magnitude of this association was comparable when taking into account BMI in the second model (*β* = −0.18, 95% CI: -0.31, -0.04). When adjusting for smoking status, the association was lowered to -0.17 (95% CI: -0.30, -0.03). We also modeled CMV antibody as a continuous variable and found that one unit increase of CMV antibody was associated with -0.06 (95% CI: -0.11, -0.01) decrease of leukocyte TL when adjusting for age, sex, BMI, and smoking status in the third model. We tested the interaction term between sex and CMV antibody and did not find statistical significance.

Likewise, we studied the association of CRP with leukocyte TL and presented the results in Table 3. The same modeling strategies were used as CMV antibody described above. In Model 3, after controlling for age, sex, BMI, and smoking status, people in the highest group of CRP levels had -0.22 (95% CI: -0.33, -0.11) lower levels of leukocyte TL compared with those in the lowest group of CRP levels. One unit increase of CRP antibody was associated with -0.06 (95% CI: -0.11,-0.01) decrease of leukocyte TL when controlling for age, sex, BMI, and smoking status in the third model.

In Table 4, we showed the results of the joint analyses of CMV antibody and CRP with leukocyte TL. We found that the associations of CMV antibody and CRP with leukocyte TL were independent of each other. The effect magnitudes were similar to those in Tables 2 and 3. We did not find a significant interaction effect of CMV antibody and CRP.

## 4. Discussion

In the present study, we tested the hypothesis that previous CMV infection (measured by antibody) was associated with shorter leukocyte TL and this relationship might be modulated by CRP. We investigated this association in a large cohort, which is a population-based survey using the NHANES data. We found that higher levels of previous CMV infection were associated with shorter leukocyte TL. This observed association only slightly changed after controlling for multiple covariates including age, sex, BMI, and smoking status. Additionally controlling for CRP in the multivariable regression models did not change the effect size too much, which might suggest that previous CMV infection influences leukocyte TL through biological pathways beyond CRP.

Leukocyte TL has been reported to be a predictor for various aging-related diseases and to be influenced by stress and inflammation. However, its association with infection, particularly CMV infection, has seldom been investigated. Addressing these associations is of significance in enhancing our knowledge of how the underlying mechanisms could affect different aspects of immune and biological aging. A previous clinical study reported that previous CMV infection could induce a strong decrease in T cell TL [18]. However, the Berlin BASE-II study performed a similar comparative analysis of leukocyte TL in CMV-positive and CMV-negative individuals and only found leukocyte TL to be slightly longer in CMV-negative participants (*P* = 0.056) [20]. This study did not perform multivariable analyses to control the potential confounding factors. A recent study using data from the Baltimore Longitudinal Study on Aging employed sophisticated models with adjustment for more confounders and reported that CMV infection was associated with shorter leukocyte TL [19]. Studies examining the association of CRP with leukocyte TL were more than that of CMV infection. Most of these studies observed shorter TL to be related to higher CRP levels [23–25]. A recent Mendelian randomization study using CRP genetic variants suggested that CRP could a causal risk factor for TL [26]. Our analyses were largely comparable to these results, but with larger sample sizes.

Previous studies also reported inflammation was associated with CMV infection [27, 28]. It is thus natural to hypothesize that inflammation (measured by CRP) could moderate the associations between previous CMV infection and leukocyte TL. In our study, we examined this hypothesized relationship. However, the findings did not support this hypothesis. When adjusting for CRP in the regression models, the magnitude of the observed association was attenuated slightly. This implies that CRP might not be an important mediator for the association of previous CMV infection and TL. The influence of previous CMV infection on shorter TL might be through other pathways.

Several strengths and limitations for the present study should be acknowledged. First, leukocyte TL was processed and assessed in a lab using well-established PCR methods. Second, the population recruited in this study were chosen as a random sample by NHANES. The sample size was larger compared with previous studies and could be representative of the age span of the participants studied, which allowed the generalization. Lastly, we adjusted for a number of potential confounders in the multivariable regression analyses including age, BMI, smoking status, and sex. These results in general suggest that our observed association for previous CMV infection and leukocyte TL was not dependent of the studied potential confounding variables. However, a few limitations should also be noted. The design of our cross-sectional study makes it hard to infer causality because of the limitations of the data collection. In this study, all these biomarkers were measured from the blood that was collected at the same time. Therefore, it is difficult to make difference between the causes and consequences. Additionally, there might be other variables that could potentially confound the observed association that we did not control. Residual confounding should be taken into account of or adjusted for in either the study design or analysis stage in future studies.

In summary, our present study demonstrates that previous CMV infection was associated with shorter leukocyte TL in the study participants of NHANES and this association was independent of CRP, suggesting that previous CMV infection may affect TL through other pathways. Our analyses could shed light on the underlying biology of immune function and its roles in biological aging.

## Figures and Tables

**Table 1:** Basic characteristics of study participants in NHANES.

Variables	Men (*n* = 1816)	Women (*n* = 2171)
Age	35.1 (0.3)	35.0 (0.3)
Telomere length (*T*/*S* ratio)	1.12 (0.02)	1.13 (0.02)
Cytomegalovirus antibody (AU/mL)	1.09 (0.04)	1.48 (0.03)
Body mass index (kg/m^2^)	27.6 (0.2)	27.9 (0.2)
C-reactive protein (mg/dL)	0.29 (0.01)	0.45 (0.02)
Smoking	51.0%	42.1%
Ethnicity		
Non-Hispanic White	10.1%	8.4%
Non-Hispanic Black	7.1%	8.1%
Mexican American	68.7%	67.8%
Others	14.1%	15.7%

**Table 2:** Association between cytomegalovirus antibody and telomere length in NHANES.

Cytomegalovirus antibody	Model 1 (crude model)	Model 2 (age, sex adjusted model)	Model 3 (multivariable adjusted model)
Q1	0 (reference)	0 (reference)	0 (reference)
Q2	0.03 (-0.07, 0.13)	0.04 (-0.05, 0.14)	0.04 (-0.05, 0.14)
Q3	-0.04 (-0.18, 0.10)	-0.04 (-0.17, 0.10)	-0.03 (-0.17, 0.10)
Q4	-0.18 (-0.32, -0.05)	-0.18 (-0.31, -0.04)	-0.17 (-0.30, -0.03)
Continuous	-0.07 (-0.12, -0.01)	-0.07 (-0.12, -0.01)	-0.06 (-0.11, -0.01)

Q: quartile.

**Table 3:** Association between C-reactive protein and telomere length in NHANES.

C-reactive protein	Model 1 (crude model)	Model 2 (age, sex adjusted model)	Model 3 (multivariable adjusted model)
Q1	0 (reference)	0 (reference)	0 (reference)
Q2	-0.10 (-0.19, 0.001)	-0.08 (-0.17, 0.01)	-0.08 (-0.17, 0.01)
Q3	-0.17 (-0.35, 0.01)	-0.13 (-0.29, 0.02)	-0.13 (-0.29, 0.03)
Q4	-0.28 (-0.42, -0.15)	-0.23 (-0.34, -0.11)	-0.22 (-0.33, -0.11)
Continuous	-0.08 (-0.12, -0.03)	-0.06 (-0.10, -0.02)	-0.06 (-0.09, -0.02)

Q: quartile.

**Table 4:** Joint association of cytomegalovirus antibody and CRP with telomere length in NHANES.

Variables	Model 4 (additionally CRP-adjusted model)
*Cytomegalovirus antibody*	
Q1	0 (reference)
Q2	0.04 (-0.05, 0.14)
Q3	-0.03 (-0.17, 0.11)
Q4	-0.16 (-0.29, -0.02)
Continuous	-0.06 (-0.11, -0.01)
*C-reactive protein*	
Q1	0 (reference)
Q2	-0.07 (-0.17, 0.02)
Q3	-0.13 (-0.29, 0.04)
Q4	-0.25 (-0.32, -0.11)
Continuous	-0.06 (-0.09, -0.02)

Q: quartile.

## Data Availability

The data were provided in the supplementary file.

## Supplementary Materials

Supplementary Materials This supplementary file contains the full original data analyzed in this article. Each row refers to one study participant and each column refers to one variable.
